# *Debaryomyces hansenii*: an old acquaintance for a fresh start in the era of the green biotechnology

**DOI:** 10.1007/s11274-022-03280-x

**Published:** 2022-04-28

**Authors:** Clara Navarrete, Mònica Estrada, José L. Martínez

**Affiliations:** 1 Department of Biotechnology and Biomedicine, Technical University of Denmark, Søltofts Plads Building 223, 2800 Kgs. Lyngby, Denmark; 2 Section of Synthetic Biology (DTU Bioengineering), Department of Biotechnology and Biomedicine, Technical University of Denmark, Søltofts Plads Building 223, 2800 Kgs. Lyngby, Denmark

**Keywords:** Non-conventional yeast, Green transition, Fermentation, Omics technologies, Waste revalorization, Salt tolerance

## Abstract

The halophilic yeast *Debaryomyces hansenii* has been studied for several decades, serving as eukaryotic model for understanding salt and osmotic tolerance. Nevertheless, lack of consensus among different studies is found and, sometimes, contradictory information derived from studies performed in very diverse conditions. These two factors hampered its establishment as the key biotechnological player that was called to be in the past decade. On top of that, very limited (often deficient) engineering tools are available for this yeast. Fortunately *Debaryomyces* is again gaining momentum and recent advances using highly instrumented lab scale bioreactors, together with advanced –omics and HT-robotics, have revealed a new set of interesting results. Those forecast a very promising future for *D. hansenii* in the era of the so-called green biotechnology. Moreover, novel genetic tools enabling precise gene editing on this yeast are now available. In this review, we highlight the most recent developments, which include the identification of a novel gene implicated in salt tolerance, a newly proposed survival mechanism for *D. hansenii* at very high salt and limiting nutrient concentrations, and its utilization as production host in biotechnological processes.

## Introduction

*Debaryomyces hansenii* is a halotolerant and xerotolerant non-conventional yeast with an enormous potential to be used in biotechnology (Navarrete and Martínez 2020; Prista et al. 2016). In recent years, the interest in *D. hansenii* has increased again and several studies have contributed to a better understanding of its halotolerant/halophilic behavior, using new methodology (such as controlled bioreactions) and omic-techniques (Navarrete et al. 2021a, b). Furthermore, new and interesting physiological characteristics have been described, presenting this yeast as an excellent option when working in harsh conditions that are commonly found in diverse industrial processes (Navarrete et al. 2021a, b).

Environmental technology or green technology are terms used to define a type of science that seeks for more environmentally friendly methods to e.g. produce different compounds, while reducing the negative impact of those processes on the environment. For instance, this non-conventional yeast is able to grow in media containing high concentration of salt, meaning that the use of pure water sources will not be necessary when industrially growing this yeast. Along this line, a recent work described the higher resistance of *D. hansenii*, and other halotolerant yeasts, to organic solvents/reagents in seawater rather than in freshwater (Andreu and del Olmo 2020), which indicates the possibility of using this yeast as biocatalysts in seawater. In the case of *D. hansenii*, its ability to grow in media containing high concentration of salt means that the use of pure water will not be necessary for the industrial production of biomass. Additionally, the utilization of high salt concentrations decreases the risk of contamination and reduces the costs due to sterilization (Navarrete et al. 2020; Navarrete and Martínez 2020). Moreover, the ability of *D. hansenii* to utilize different carbon sources and to growth in the presence of certain inhibitors, makes it an excellent option when working in industrial bioprocesses based on both lignocellulosic and non-lignocellulosic biomass feedstocks (Dyerberg et al. 2022).

## Engineering *D. hansenii*

**Fig. 1:**
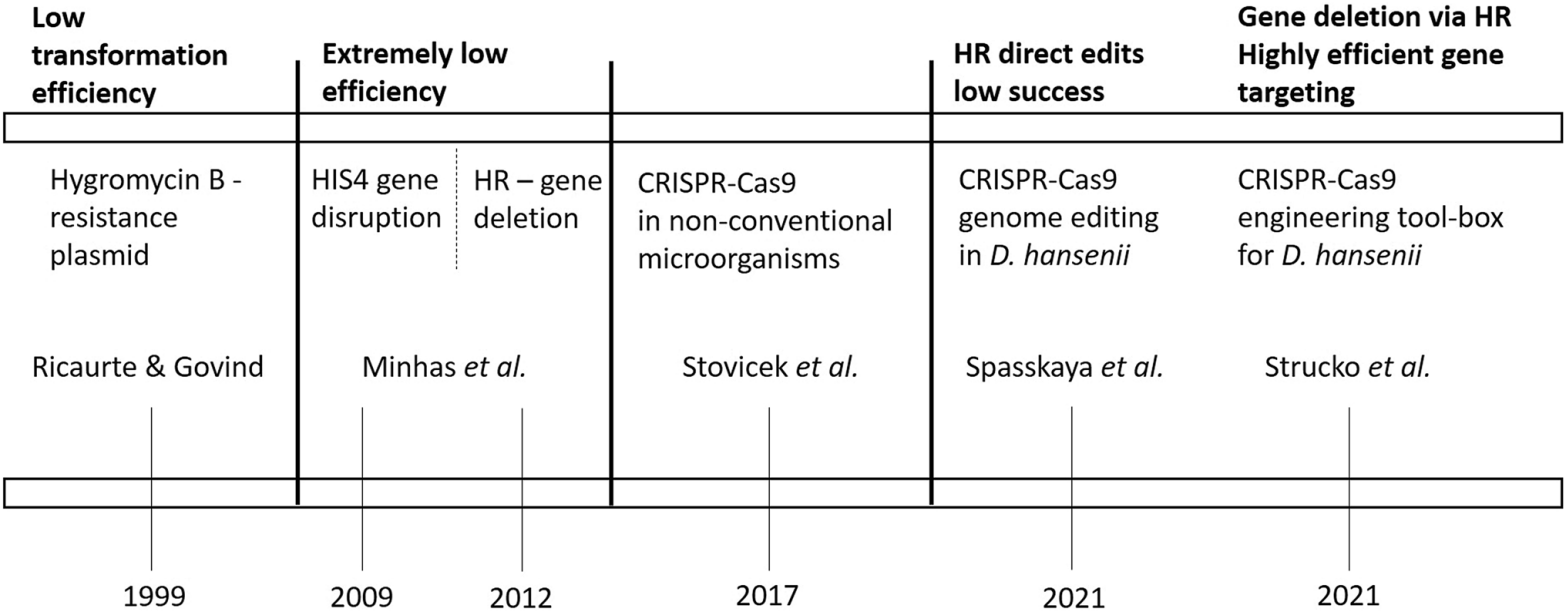
Chronological scheme of the different tools used to engineer *D. hansenii* in the last decades, their main characteristics and possible shortages

For decades, the only limitation when working with *D. hansenii* was the lack of efficient and reproducible molecular tools allowing engineering of this yeast. Consequently, for many years, halotolerance studies in *D. hansenii* were carried out using heterologous expression in *Saccharomyces cerevisiae* (Prista et al. 2016). Although several research groups have been successful in developing some molecular tools, it is in the twenty-first century when the application of novel technologies has made possible highly efficient transformation methods and the obtaining of knockout strains of *D. hansenii* (Fig. 1). These are important steps to improve both our general knowledge about the yeast, and to obtain better host strains for their future use in the industry.

### Tools developed in the early 2000s

The first documented attempt to engineer *D. hansenii*, was performed by Ricaurte and Govind (1999). They demonstrated *D. hansenii’s* sensitivity to hygromycin B and transformed the yeast with plasmids containing a hygromycin B resistance gene. However, in these experiments the transformation efficiency was very low. Genome integration was also achieved by the same authors, who used integrative plasmids with the same dominant marker (Ricaurte and Govind 1999).

A system based on *HIS4* as auxotrophic marker was also effectively used for gene disruption in *D. hansenii* (Minhas et al. 2009). The authors were able to delete three different genes (*DhXR*, *DhPPZ1* and *DhMPK1*) by homologous recombination, though the gene disruption method showed again an extremely low efficiency (only 4/25 clones showing the desired phenotype) (Minhas et al. 2009, 2012).

In *S. cerevisiae*, the preferred method for double-strand breaks repair is HR (homologous recombination). On the other hand, many other yeast species prefer the NHEJ (non-homologous end joining) pathway, including *D. hansenii*. In order to avoid random genome integration and the consequent difficulty to find correct mutant strains, the adopted strategy to generate NHEJ-deficient mutants seems to be the key to easily obtain knockout strains of *D. hansenii* (Krappmann 2007).

### The use of novel techniques: CRISPR-Cas9 to engineer non-conventional yeasts

The discovery of CRISPR-Cas9 technology and its applications in genome editing, have had a great impact on the engineering and synthetic biology fields, for *S. cerevisiae* but also for many other microorganisms, including some non-conventional yeast (Stovicek et al. 2017). As mentioned before, genetic engineering of this yeast has been highly limited mostly due to the NHEJ pathway as the preferred one by *D. hansenii*. For this reason, it is nearly impossible (or highly inefficient) to introduce genome alterations via homology based gene targeting in this yeast (Krappmann 2007; Spasskaya et al. 2021; Strucko et al. 2021).

A recent study described the successful application of CRISPR/Cas9 in *D. hansenii* genome editing for the first time (Spasskaya et al. 2021). However, it was mainly used for gene inactivation via defective NHEJ repair, while homologous recombination (HR) directed edits had still low success.

Shortly after the aforementioned study, Strucko et al. (2021) reported the development of a highly efficient CRISPR-Cas9 engineering toolbox for *D. hansenii*, which allows introducing precise Cas9-mediated point mutations and marker-free gene deletion via HR. In this work, the authors disrupted the NHEJ pathway in *D. hansenii* creating a mutant for the gene *KU70,* which codes an essential protein for NHEJ repair. Therefore, a highly efficient gene targeting by HR was achieved in this mutant strain. Besides, a CRISPR-Cas9 tool, compatible with multiplex gene targeting in *D. hansenii* was also successfully developed (Strucko et al. 2021).

## First *D. hansenii*´s characterization in controlled environments

*D. hansenii* has been traditionally used as a model organism to study osmotolerance and salt tolerance in eukaryotic systems (Adler et al. 1985; Prista et al. 1997, 2005). Therefore, the role of potassium and sodium fluxes on ion homeostasis has been extensively studied on this yeast (Almagro et al. 2001; García-Salcedo et al. 2007; Martínez et al. 2011; Montiel and Ramos 2007; Prista et al. 2007; Velkova and Sychrova 2006). However, it was difficult to find consensual information on behavioral patterns of this yeast due to the diverse culture conditions (e.g. media composition, pH, temperature, strain type, etc.) and different experimental methodologies used by the different research groups.

*D. hansenii* has been already used to produce industrially-relevant products such as xylitol, trehalose and fat-soluble vitamins (Navarrete and Martínez 2020), that the yeast is able to produce naturally. Specially, the xylitol market in food and pharmaceutical industry has exponentially increased during the last decades (Musatto 2012). Several authors have published their work about increased xylitol production by *D. hansenii* during glucose, glycerol or xylose fermentation. For instance, a 2.5-fold increased xylitol concentration was obtained when the *XDH* gene, coding for a xylitol dehydrogenase, was disrupted in *D. hansenii* (strain CBS767) (Pal et al. 2013). The authors also observed better xylitol yields when the mutant cells were grown with glycerol as co-substrate during xylose fermentation in flasks and in bioreactors. Another study, investigated the use of rapeseed straw hemicellulosic hydrolysate as a fermentation medium for xylitol production by *D. hansenii* in flasks (López-Linares et al. 2018). More specifically, the authors obtained 0.45 g/g of xylitol without the need to completely eliminate the toxic compounds generated during the conversion of xylose to xylitol. This work also revealed a negative impact of glucose on the yeast performance along the process. All these studies are of extreme relevance when introducing *D. hansenii* as a suitable host for biotechnological processes, but none presented a complete characterization of the yeast during the fermentation process. The majority of them were performed in shake flasks (non-controlled conditions) and exclusively focused on the specific processes but not on the cell physiology under the production conditions.

The first characterization of *D. hansenii* in batch cultivation and under highly controlled lab-scale bioreactors was reported by our lab in 2021 (Navarrete et al. 2021a). In this work, we contributed to a more complete picture of the central carbon metabolism and the external pH influence on *D. hansenii*´s ability to tolerate high salt concentrations (Na^+^ and K^+^) (Navarrete et al. 2021a). Furthermore, a differential effect of both salts was observed, with NaCl exhibiting a more significant positive impact than KCl. Besides, higher growth rates at a combination of low pH and high salt vs. low pH and no salt were also described.

**Fig. 2:**
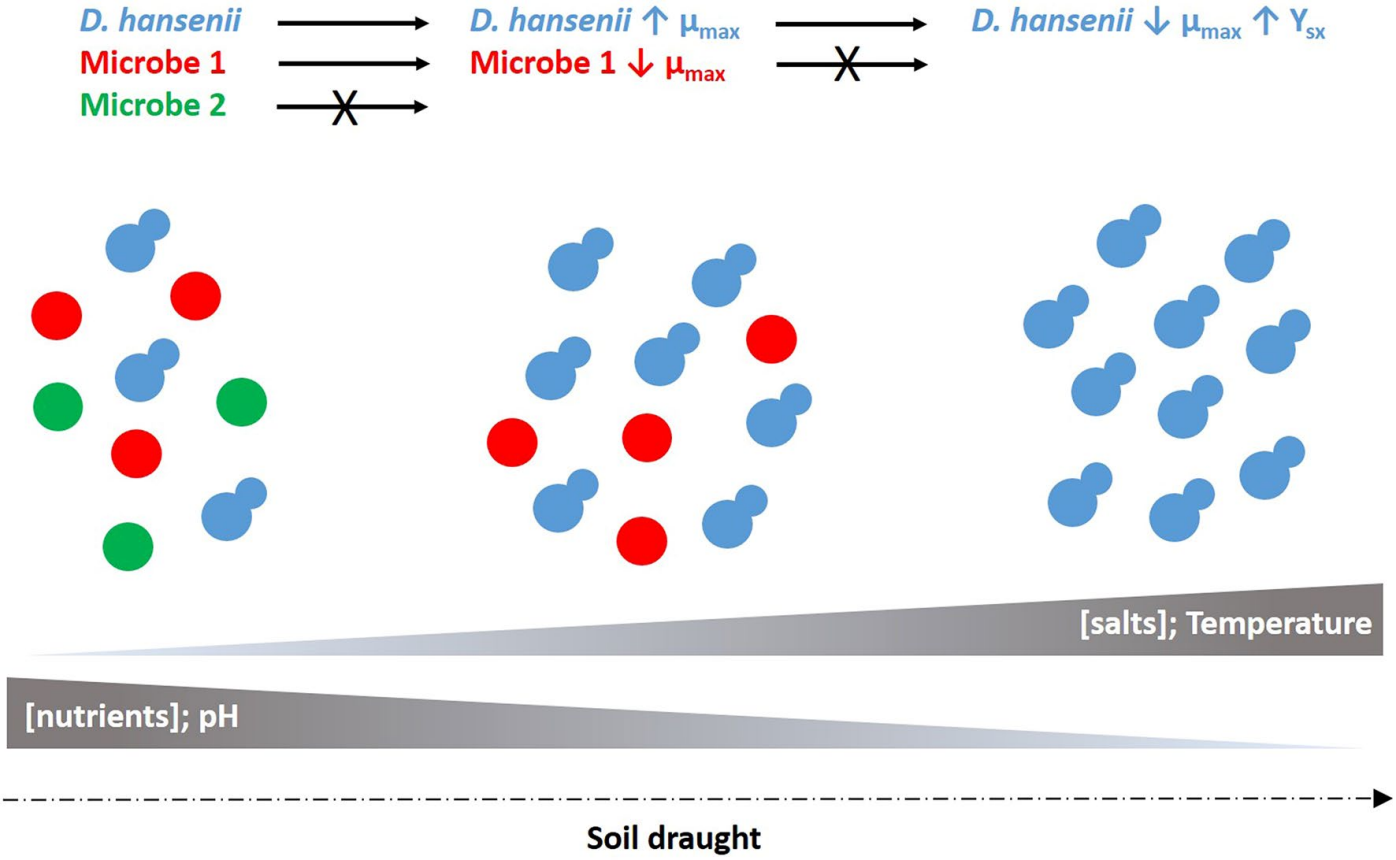
Graphic illustration of a novel survival strategy for *D. hansenii* growing in environments with very high salinity, proposed by Navarrete et al. (2021a)

Additionally, this study led us to propose a novel survival strategy for this yeast, at very high salinity (Fig. 2). We demonstrated that *D. hansenii* increases its growth rate at moderate-high salinity (over 1 M) which permits metabolizing the most of the available carbon quite rapidly in this conditions, thus limiting resources for other competitors (Navarrete et al. 2021a). When the salt concentration increases to higher levels (2 M) in the environment, the yeast cells slow down their growth rate while still proliferating and at the same time increasing the carbon yield conversion into biomass. That allows overpopulating areas rich in salts, for example thawing Arctic glacier and coastal environments where *D. hansenii* has been described to be the most prevalent species (Butinar et al. 2011; Jacques et al. 2015).

## Discovery of novel genes/proteins involved in *D. hansenii*´s response to high salt

Several studies touching upon *D. hansenii*´s metabolism at a molecular/regulatory level have been published in the last 20 years (Gori et al. 2006; Martínez et al. 2012; Papouskova and Sychrova 2007; Ramos-Moreno et al. 2019). However, studies covering proteomics and transcriptomics analyses under salt exposure were very limited in number and difficult to consensuate, due to the utilization of different strains, experimental conditions or sampling regimes among them.

Particularly, a couple of proteomics analyses identified a number of induced proteins involved in the glycerol synthesis and the upper part of the glycolysis, in the presence of NaCl. Examples of repressed proteins were e.g. those related to the lower part of the glycolysis, Krebs cycle or aminoacids biosynthesis (Gori et al. 2006). Martínez et al. (2012) also studied *D. hansenii*´s proteome but in the presence of KCl, correlating very well their observations with the results previously presented by Gori and collaborators.

A key factor when investigating the effect of high salt on *D. hansenii*´s metabolism is the cultivation time. Some authors have described high sodium concentrations as detrimental after only 2–3 h of exposure, whereas it has been recently confirmed that longer cultivation times are needed in order to observe the overall positive effect of salts on the cell metabolism of this yeast (Navarrete et al. 2021a; Papouskova and Sychrova 2007).

The most recently published study on *D. hansenii* show a holistic and integrative view, containing several –omics analysis (proteomics, phosphoproteomics and RNAseq analyses). This study was performed under 1 M NaCl/KCl using chemostats (continuous cultivations), a cultivation setup which overcomes the limitations normally observed in batch processes. Chemostats ensure that all the cells in the culture are in steady-state exponential growth and therefore, the different culture conditions are fairly comparable. This means that all the cells can be set at the same growth rate by controlling the feeding in and out the bioreactor (Navarrete et al. 2021b). The main conclusion from this work was that wider, non-specific and less coordinated expression changes occurred when KCl was used, and in contrast a more specific and targeted response occurred in the presence of sodium. Furthermore, from the transcriptomics and global proteomics analyses, an increase in the respiratory metabolism was observed in the presence of both salts that correlates with a higher growth rate and better cell performance. Additionally, several proteins related to the cellular responses to oxidative stress are more abundant or increased their translation under these conditions (e.g. Sod1, Ctt1, Srx1), which can also be linked with a higher respiratory metabolism stimulated by the presence of salt. On top of this, resistance to drugs and growth inhibitors was also observed by the overexpression of genes involved in this type of response (e.g. *TPO1* and *PDR5*) (Navarrete et al. 2021b). An interesting observation in this study was that, among all the responsive genes and proteins to the presence of salt, around 40–50% of them are still uncharacterized or have yet unknown function.

Interestingly from the phosphoproteomics analysis, a novel and yet uncharacterized transmembrane transporter (B5RUG0) was identified. It seems to be involved in the response to high salt concentrations, and its aminoacid sequence shows some homology with a certain membrane transporter involved in metal tolerance in *Candida* species. This specific transporter showed to be highly regulated in *D. hansenii*, which makes it a really interesting target of study and therefore worth to be further investigated (Navarrete et al. 2021b).

## The era of the high-throughput (HT) analyses and their potential use in the study of *D. hansenii*

### The discovery and implementation of the HT systems in the recent years

Since the introduction of molecular biology, a tool to develop hundreds of engineered cells in a short period of time has always been in the wish list of microbial engineers. In recent years, the advances in genetic engineering and the discovery and implementation of advanced techniques such as CRISPR/Cas and Multiple Automated Genome Evolution (MAGE) have allowed the generation of large libraries, overcoming this challenge (Guo et al. 2018; Vervoort et al. 2017). Fortunately, even newer ones have been created that allow the generation of a huge amount of engineered cells in a very short time, thus creating the need to identify and select the best performing strain or the one with the desired phenotype in the shortest amount of time and with the lowest cost possible. Recently, High-Throughput Screening (HTS) methodologies are gaining interest as crucial systems for this purpose (Sarnaik et al. 2020).

HTS techniques allow scaling-down the assays to the μL or mL scale using microtiter plates to test as many conditions as possible in a reasonable timeframe, gaining insight into the process (Xiao et al. 2015). These plates can, for example, act as micro-reactors and be introduced in automated microbioreactor systems (e.g. BioLector™) that can simulate industrial-scale fermentations, monitoring critical parameters such as optical density for biomass determination and temperature, among others (Zeng et al. 2020). Moreover, each well can also act as a miniature test tube in which multiple analysis can be performed, as ELISA or enzymatic assays (Boettner et al. 2002; Du et al. 2019; Weinberger et al. 2020), reducing the amount of chemical reagents and sample used. Then, the plates can be placed into microplate readers (e.g. SpectraMax ID3 or CLARIOStar Plus) which have different measurement modes as absorbance, fluorescence, and luminescence (Kodedová and Sychrová 2016; Kamli et al. 2021). Most of these microbioreactor systems, as the aforementioned BioLector™, can be used to design optimal bioreaction setups. The de-novo design of suitable fermentation media can be afterwards upscale for industrial production volumes with a high degree of reproducibility (Jacobsen et al. 2020). Some relevant examples currently available in the literature are summarized in Table 1.

**Table 1:** List of the most representative equipment used as HTS and their function

Function	Equipment	Commercial house	References
Equipment for HTS			
Microplate reader	SpectraMax ID3	Molecular devices	Ismail et at. (2021), Hartmann et al. (2021)
	CLARIOStar Plus	BMG LABTECH	Almeida et al.(2021), Chapman et al. (2021)
Microbioreactors	BioLector II	M2p-labs	Back et al. (2016), van Dijk et al. (2020)
	Ambr250 Cell Culture	Sartorius	Manahan et al. (2019), Eng et al. (2021)
Equipment to automate HTS methods			
Colony picker	Qpix 400	Molecular devices	Varberg et al. (2020), Zhang et al. (2021)
	ROTOR	Singer Instruments	Dyerberg et al. (2022), Aguiar-Cervera et at. (2019)
Liquid handling robots	OT-2	Opentrons	Tenhaef et al. (2021), Biedermann et al. (2022)
	TECAN	Tecan	Machillot et al. (2018), Cavallo et al. (2020)
	Hamilton	Hamilton	Weinberger et al. (2020), Benieyton et al. (2016)

Nevertheless, plate handling and pipetting for the screening of thousands of clones or conditions is a very demanding work. Hence, the development of automated systems to facilitate this task have made HTS to become a feasible reality (Burckhardt 2018; Sarnaik et al. 2020). Some companies have designed advanced robots to automate entire workflows and laboratory protocols (Table 1). The advantages of these novel technologies compared to traditional handling are e.g. (i) they can perform repetitive tasks with lower error distribution, which reduces human labor and ensures reproducibility; (ii) they can eliminate cross-contamination; and (iii) they can reduce the operator’s risk of handling toxic substances (Bogue 2012; Strimatis 1989).

Finally, with the large amount of data that HTS systems generate, it is necessary to develop new computational techniques to analyze and make predictions from the results (Leavell et al. 2020). Hence, the future of HTS platforms is to include modelling and Machine Learning approaches to further optimize the screening. Along this line, recent studies have shown the beneficial impact of using Machine Learning algorithms decreasing the number of experiments needed to reach a satisfactory conclusion (Dreiman et al. 2021; Simmons et al. 2008).

### Using advanced HT technologies for screening a library of D. hansenii for strains with higher potential for industrial production setups

The implementation of HTS has contributed to a better understanding of *D. hansenii* metabolism. Recently, a study conducted in our lab analyzed the positive effect of sodium ions on the performance of a selected *D. hansenii* array from the DTU Bioengineering Fungal collection, and their capabilities to tolerate abiotic stresses compared to the model strain most commonly used in published studies during the last three decades (Dyerberg et al. 2022). Using advanced robotics, we were able to screen up to 61 strains of *D. hansenii* in more than 50 different media conditions.

In this study, we analyzed the growth of the yeast strains in agar plates comparing eight different carbon sources, under the presence of increasing concentrations of fermentation inhibitors often released from pretreatment of complex feedstocks (e.g. vanillin, furfural or hydroxymethyl-furfural (HMF) among others), and with the presence or absence of NaCl. We used a ROTOR (Singer Instruments) robot to spot the yeast library from liquid to agar plates (128 colonies per plate), and a PhenoBooth (Singer Instruments) to monitor the colonies growth after 48 and 144 h. The diameter and density of the colonies were used as an indicator of carbon utilization and stress tolerance. Moreover, the effect of sodium ions on the strain’s behavior was studied when growing in medium with low pH. A final test was performed in a microbioreactor (BioLector II, mp2 Labs) to assess growth stability and determine the differences in growth rates (Dyerberg et al. 2022).

The results obtained concurred with those previously observed by Prista et al. (2016) and Navarrete et al. (2021a): not only *D. hansenii* can thrive at high osmotic pressure, but also sodium ions have a protective effect on its growth under the presence of other stress factors, improving its performance. We additionally found that adding 1 M NaCl to the media enhanced the assimilation of pentose sugars, such as arabinose and xylose, and relieved the abiotic stress caused by the presence of some fermentation inhibitors tested, such as furfural or HMF. Moreover, we noticed a positive effect on cell growth in acidic environments when sodium was present, as Navarrete et al. (2021a) showed in their previous work.

With this screening, Dyerberg et al. (2022) demonstrated that *D. hansenii*´s behavior with sodium ions is not strain-dependent, but it is extended to the species level. Interestingly, some strains showed an increased favourable phenotype, that allowed to select the most interesting ones for their further study and potential utilization in production processes at industrial volumes.

## Biotechnological use of *D. hansenii* in waste-streams revalorization processes

*D. hansenii* has a huge potential as a new cell factory for the revalorization of industrial waste streams (Kaur et al. 2021; Navarrete et al. 2020). The inherent capabilities that this non-conventional yeast presents–the halophilic character, the high osmotic tolerance and the low pH resistance, among others–allow it to grow using complex feedstocks instead of the traditional commercial defined media, as lignocellulosic biomass or industrial by-products (Capusoni et al. 2019; Chao et al. 2009).

Lignocellulosic biomass, including wood, agricultural, forestry, food and municipal solid waste, is currently employed as a substrate by the biorefinery industry for the production of biofuels, organic acids, and other valuable chemicals (Berlowska et al. 2018). It is the most abundant renewable raw material in the world as it is not season dependent and moreover, it contains high concentration of sugars as hemicellulose, cellulose and lignin carbohydrate polymers (Singhvi and Gokhale 2019). Nevertheless, due to the recalcitrant nature of the lignocellulosic biomass, it is necessary to perform a chemical or biological pretreatment to release the fermentable sugars from the polymers and make them accessible for the microorganisms, which imposes the main challenge for its industrial use. During this process, some microbial growth inhibitors can also be released depending on the hardness of the conditions used and the biomass source. For example, some fermentation inhibitors often released from acid or alkaline hydrolysis pretreatment are phenolic compounds like vanillin, furan aldehydes such as furfural or HMF, and organic acids including formic acid or acetic acid (Bhatia et al. 2020). To avoid it, alternative pretreatment strategies are also available, such as enzymatic pretreatments (Hosseini Koupaie et al. 2019).

*D. hansenii*, unlike the model organism *S. cerevisiae*, is able to metabolize a broad range of sugars usually found in lignocellulosic biomass, including arabinose, mannose, or xylose (Dyerberg et al. 2022). It can also grow when some inhibitors are present in the environment. For example, it tolerates up to 4 g/L of furfural and 2 g/L of HMF, and this tolerance increases when sodium is present in the media (Dyerberg et al. 2022). The capacity to thrive under harsh conditions makes *D. hansenii* a perfect candidate for the lignocellulosic biomass revalorization. As an example, Portilla et al. (2008) used *D. hansenii* to revalue vine-shoot trimming waste and convert the contained xylose to xylitol. However, more studies are needed to gain further insights on the process involving *D. hansenii* and lignocellulosic biomass.

Other “dirty” feedstock sources are potential substrates for *D. hansenii*. Some industrial waste-derived streams, as the ones coming from the food industry, can still be rich in nutrients and are a perfect example of a possible feedstock source. More specifically, the dairy industry produces vast amounts of wastewater on a daily basis that contains proteins, salt, fatty substances, lactose and chemicals used during the cleaning process of the milk (Arvanitoyannis and Giakoundis 2006). Usually, these effluents cannot be discharged in wastewater plants because they exceed the permitted contaminant limits. Consequently, companies are searching for alternatives to treat this waste and reduce the levels of chemical and biological contaminating factors, or revalorite it. The superior performance of *D. hansenii* under high salinity and high osmotic pressure environments, and its capacity to metabolize lactose, could make it a suitable cell factory for this purpose. On top of that, as many microorganisms cannot survive these stressful conditions, the yeast would have a significant advantage to overcompete other microorganisms, which could spare the sterilization needs, hence reducing the operational costs. Moreover, using the newly established genetic engineering tools for *D. hansenii* (Spasskaya et al. 2021; Strucko et al. 2021) it may now be possible to produce high-value bioproducts from waste by introducing heterologous biosynthetic pathways in this yeast.

## Future perspectives

The challenges to overcome, derived from the development of novel bioreaction processes for the so-called green transition, require implementing novel microbial cell factories with higher robustness. These novel cell factories must show an innate better tolerance to industrial fermentation conditions whilst not compromising their production yields or productivity. Normally, increasing the tolerance in the classical hosts require numerous rounds of engineering that negatively affect these two important parameters (Navarrete and Martínez 2020).

In this regard, *D. hansenii* possesses all the advantages to become a suitable cell factory for the green biotechnology revolution. One of the main challenges faced by most industries is the exorbitantly high amount of waste generated every day which, if uncontrolled, can cause environmental and health damage (Donzella et al. 2021; Klitkou and Bolwig 2019; Liguori and Faraco 2016). Therefore, exploiting the capacity of *D. hansenii* for waste-streams revalorization could help to achieve a more sustainable waste management, switching from a linear to a circular economy. High-value chemicals, food ingredients such as vitamins and antioxidants, or recombinant proteins such as industrial enzymes or peptide-based therapeutics, could be produced from the use of these waste-streams. Until very recently, *D. hansenii’s* use for industrial production was limited to flavonoids or xylitol, which were naturally produced by this yeast. However, with the recently demonstrated capability to introduce recombinant biosynthetic pathways, the production possibilities are uncountable.

It is important to stress the fact that higher levels of understanding of *D. hansenii’s* metabolism are still necessary, as the latest advances in terms of physiological/molecular characterization simply scratched the surface: *Debaryomyces*’ potential still remains to be unfolded at its full. For example, as previously mentioned, over 40–50% of salt responsive genes and proteins remain uncharacterized, and little is known about the regulatory networks that govern the positive responses in terms of cell performance to harsh environments (high salt / low pH / low carbon).Therefore, the development of a comprehensive genome scale metabolic model to better understand metabolic fluxes and integrate multi-omics analyses would definitely help to achieve significant advances. In this sense, the follow up development of high precision engineering tools based on CRISPR/Cas9, and the implementation of advanced HTS / engineering methodologies will play an important role in the near future. We are now able to introduce non-natural biosynthetic pathways into *D. hansenii’s* and, most importantly, from now onwards we have the possibility to obtain a library of mutants, that will be paramount to better understand and characterize the aforementioned unknown genes / proteins responsive to salt. This will ultimately help to design better production strains while increasing our understanding on this yeast’s peculiar and interesting behavior.
